# Development of maize‐based instant porridge flour formulated using sweet lupine, orange‐fleshed sweet potato, and moringa leaf powder

**DOI:** 10.1002/fsn3.4483

**Published:** 2024-09-25

**Authors:** Tsiyonemariam Nega Dessta, Zemenu Kerie Terefe

**Affiliations:** ^1^ Department of Food Engineering Dilla University Dilla Ethiopia; ^2^ School of Nutrition, Food Science and Technology Hawassa University Hawassa Ethiopia

**Keywords:** extrusion, instant porridge, maize, malnutrition, moringa, orange‐fleshed sweet potato, protein, sweet lupine

## Abstract

Malnutrition and food insecurity are major public health problems in developing countries including Ethiopia. Because of an economic issue and information gap, developing countries' survival depends on foods rich in carbohydrates but deficient in protein and micronutrients. However, it is paramount to mix ingredients that complement different nutritional profiles to alleviate the problem of malnutrition. Hence, in this research, maize, orange‐fleshed sweet potato, sweet lupine, and moringa leaf powder composites were used to develop nutritious instant porridge flour through extrusion cooking. Formulations containing powdered maize, orange‐fleshed sweet potato, sweet lupine, and moringa leaf were developed in the following ratios: 100:0:0:0, 50:35:10:5, 45:30:15:10, and 40:25:20:15, respectively. A completely randomized design was used to determine the blending effect on the functional properties, proximate composition, minerals, and beta‐carotene content of formulated instant porridge flour. Whereas, a randomized complete block design was used for the organoleptic characteristics data. Accordingly, the addition of orange‐fleshed sweet potato, sweet lupine, and moringa leaf powder to maize‐based porridge showed a significant (*p* < .05) increase in bulk density (0.70 ± 0.02 to 0.74 ± 0.03), water solubility index (10.09 ± 0.08 to 14.16 ± 0.23), protein (9.00 ± 0.00 to 21.10 ± 0.02), ash (1.43 ± 0.07 to 3.36 ± 0.08), fiber (1.55 ± 0.05 to 3.21 ± 0.01), fat (3.37 ± 0.02 to 5.37 ± 0.07), energy (370.89 ± 0.70 to 400.53 ± 0.65), vitamin A (0.00 to 41.00 ± 1.55), iron (3.49 ± 0.02 to 9.58 ± 0.29), zinc (2.89 ± 0.01 to 3.62 ± 0.06), and calcium (40.49 ± 0.42 to 113.34 ± 0.52) contents. However, water absorption index (4.76 ± 0.01 to 3.84 ± 0.01), moisture (8.50 ± 0.08 to 5.60 ± 0.04), and carbohydrate (76.11 ± 0.22 to 66.94 ± 0.02) content were decreased as a result of blending proportion compared to maize porridge (control). Formulated instant maize‐based porridge prepared from 50% maize, 35% orange‐fleshed sweet potato, 10% sweet lupine, and 5% moringa leaf powder scored the highest (above 4 out of 5 in a 5‐point hedonic scale) in all sensory attributes. In conclusion, the addition of orange‐fleshed sweet potato, sweet lupine flour, and moringa leaf powder has improved both macro‐ and micronutrients. Therefore, the development of nutritious maize‐based instant porridge formulated from readily available and underutilized crops can be achieved by extrusion cooking that helps to reduce the prevalence of malnutrition and food insecurity.

## Graphical abstract

Extrusion cooking of formulated flours significantly improves the protein content of the product. Moreover, minerals and beta‐carotene contents were enhanced as a result of formulation of different ingredients.

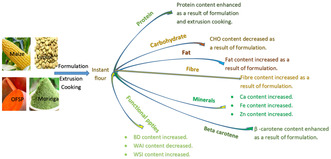

## 1 INTRODUCTION

Maize is widely grown and consumed in Ethiopia and many African countries. The demand for maize in developing countries is rising compared to both wheat and rice (Shiferaw et al., 2011). Nonetheless, maize's nutritional profile is low compared to other cereal crops; in particular, it lacks lysine, an essential amino acid. Protein–Energy Malnutrition (PEM) and dietary micronutrient deficiencies are common in sub‐Saharan Africa (SSA) as a result of the region's overdependency on starch‐dense staples like maize (Terefe et al., 2022). Sub‐Saharan Africa (SSA) continues to lead in bearing the brunt of malnutrition, and the prevalence has continued to rise to 47.3%, with the highest increases occurring in regions of Africa (Chukwu et al., 2017). The most common types of malnutrition in Ethiopia are PEM, vitamin A deficiency (VAD), iodine deficiency disorders, and iron deficiency (anemia) (Haile & Getahun, 2018). About 8.89 million children (6 months to 6 years old) are at risk of VAD in Ethiopia (Haile & Getahun, 2018). However, the government of Ethiopia has devised programs and initiatives that directly and indirectly contribute to the reduction of malnutrition through *Seqota* Declaration planned for 2016–2030 that aimed to reduce the prevalence of stunting, underweight, and wasting from 38% to 26%, 25% to 13%, and 9% to 4.9%, respectively, among children below 5 years. So, an improvement in nutrition is needed to decrease public health problems associated with Protein Energy Malnutrition (PEM) and micronutrient deficiencies. Therefore, it is advantageous to blend maize flour with other locally available ingredients that are high in protein and micronutrients to fill the lacking nutritional components in maize.

Improving the nutritional properties of foods can be achieved by adding various ingredients, especially those that are readily available but underutilized (Wandersleben et al., 2018). Legumes are classified as an important group of crop plants, and the seeds are a rich source of proteins, minerals, vitamins, and sugars (Bojňanská et al., 2012). Lupine is an underutilized legume that contains a high amount of proteins (30%–40%) with a balanced amino acid profile (Bähr et al., 2014; Habtemariam et al., 2019; Hane et al., 2017). Furthermore, it is a significant source of dietary fiber (1.6%–2.7%) and a small amount of starch that is an indispensable part of a healthy diet (Adem et al., 2020; Wandersleben et al., 2018).

Orange‐fleshed sweet potato (*Ipomoea batatas* L.) (OFSP) is a naturally biofortified crop (Kindeya et al., 2021). It is a valuable but perishable root crop in Ethiopia and is widely cultivated in the southern part of the country (Gurmu & Mekonen, 2019). According to reports from various African countries, such as Rwanda, Burundi, and Uganda, where sweet potato production is high, full adoption of OFSP could resolve VAD for 85%–95% of children who are at most risk (Haile & Getahun, 2018). Even in countries like Ethiopia that do not produce a lot of sweet potatoes, a third of the population would benefit from increased consumption of β‐carotene from orange‐fleshed sweet potatoes (Haile & Getahun, 2018; Kindeya et al., 2021).

Moringa is an indigenous multipurpose tree widely grown and cultivated in parts of Ethiopia and has tremendous nutritional and therapeutic benefits (Belay et al., 2022). Moringa leaves are poor in carbohydrates and fats but good sources of proteins (29.5%), vitamins A, B group, and C and are among the best sources of minerals, such as potassium (1933.4 mg/100 g), iron (54.8 mg/100 g), phosphorus (250.5 mg/100 g), and zinc (2.2 mg/100 g) (Belay et al., 2022; Gebretsadikan et al., 2015).

Based on the nutritional profiles, if combined with maize, all three crops offer the desired nutritional characteristics that could result in nutrient‐rich instant (extruded) food products. Pro‐vitamin A‐rich OFSP, protein‐rich lupine, and protein‐ and mineral‐rich moringa leaves are all excellent sources of these nutrients (Haile & Getahun, 2018; Bähr et al., 2014; Gebretsadikan et al., 2015; Belay et al., 2022). Hence, this research was designed to develop maize‐based instant porridge flour formulated using sweet lupine, orange‐fleshed sweet potato, and moringa leaf powder to address Protein–Energy Malnutrition (PEM) and micronutrient deficiencies in developing countries including Ethiopia.

## 2 MATERIALS AND METHODS

### 2.1 Sample collection and preparation

Ten kilograms of maize (*Melkasa_2*), 30 kg of OFSP (*Alamura variety*), and 3 kg of moringa leaves were collected from Hawassa Agricultural Research Center, southern Ethiopia. Whereas, 10 kg of sweet lupine (*Wolela* variety) was collected from the highland pulse breeding program of *Holeta* Agricultural Research Center (HARC), *Oromia* region of Ethiopia.

Maize flour was prepared, according to Zegeye et al. (2015). Maize grains were cleaned manually to remove debris and foreign matter, milled into flour in a commercial mill house, and sieved with a sieve size of 710 μm. The milled flour was packed in airtight polythene bags and stored at room temperature (22.5°C) in a dry place, until the flour was required for formulation and chemical analysis. Orange‐fleshed sweet potato flour was prepared according to Kindeya et al. (2021) and Korese et al. (2021) with slight modifications. The orange‐fleshed sweet potato was peeled using a knife after being sorted and washed with tap water. After being peeled, OFSPs were sliced using a slicer machine and blanched for 10 min at 65°C in a water bath. After draining, the treated slices were dried in an oven (binder) for 24 h at 60°C. Dried chips were milled into OFSP flour with a miller machine and sieved with a 710‐μm sieve size. The flour was packed in polyethylene plastic bags and stored at room temperature (22.5°C), until it was required for formulation and chemical analysis. The sweet lupine bean flour was prepared according to Kefale and Yetenayet (2020) and Embaby (2010) with slight modifications. About 6 kg of sample bean was soaked in water overnight. After soaking, the water was drained off and the seeds were dried at 50°C for 12 h. in the oven according to Embaby (2010). The bean was milled and sieved with a 710‐μm sieve size. The sweet lupine flour was packed in polyethylene plastic bags and stored at room temperature (22.5°C), until it was required for formulation and chemical analysis. Moringa leaf flour was prepared according to Belay et al. (2022) and Emelike et al. (2015) with slight modifications. First, the moringa leaf was sorted and washed with tap water and then dried in oven (binder) at 50°C for 12 h. The dried moringa leaves were milled using a laboratory miller and sieved through a 710‐μm sieve. The powder was then packed in polyethylene plastic bags and stored at room temperature before formulation and chemical analysis.

### 2.2 Composite flour preparation

Four formulations were prepared, which contain maize flour ranging from 40% to 100%, orange‐fleshed sweet potato flour ranging from 0% to 35%, moringa leaf powder ranging from 0% to 15%, and sweet lupine flour ranging from 0% to 20% based on the experimental layout in Table 1. Proportions were identified after a trial experiment and previous studies (Gebretsadikan et al., 2015; Kefale & Yetenayet, 2020; Zegeye et al., 2015). The goal of the trials was to increase the products' overall sensory acceptance and protein content (>15%). The composites of maize, orange‐fleshed sweet potato, moringa leaf, and sweet lupine flour were mixed using a blender.

**TABLE 1:** Proportion of maize flour, OFSP flour, sweet lupine flour, and moringa leaf powder.

Treatments	Maize flour	OFSP flour	Sweet lupine flour	Moringa leaf powder
P0	100%	0%	0%	0%
P1	50%	35%	10%	5%
P2	45%	30%	15%	10%
P3	40%	25%	20%	15%

*Note*: *p* refers to proportion.

### 2.3 Instant flour preparation

The instant flours were prepared using extrusion cooking according to Terefe et al. (2022). Extrusion was performed on a pilot scale corotating twin screw extruder (model Clextral, BC‐21 No 124, Firminy, France). The composite flours were extruded using previously optimized conditions of extrusion variables at extrusion temperature (118°C) and feed moisture (14%). Following extrusion, the products were oven‐dried for 4 h at 50°C, ground in a laboratory mill (high‐speed multifunction comminutor), and sieved using a 710‐μm sieve size. Before undergoing additional chemical analyses, the prepared extruded instant flour was placed in polyethylene plastic bags and kept at room temperature.

### 2.4 Porridge preparation

Porridge samples were prepared by modifying the method described by Akande et al. (2017) and Gebretsadikan et al. (2015). Using 200 g of each composite instant flour and 800 mL of boiling water, four distinct porridges were made in about 4 min while stirring continuously. The control sample (maize flour) of 200 g was cooked (100°C) by using 580 mL water and iodized table salt (1 g) stirring with a wooden ladle, until it attained a desirable paste consistency similar to the traditional porridge.

### 2.5 Determination of functional properties

The bulk density was calculated according to Ijarotimi and Keshinro (2012). About 5 g of flour sample was measured and transferred to a 20 mL graduated cylinder. The cylinder was repeatedly tapped until the volume remained steady. The bulk density of the sample (g/mL) was calculated as the weight of the sample per unit volume of sample (Equation 1).

(1) $$\text{Bulk density}\left(g/\mathrm{mL}\right)=\frac{\text{weight of the sample}}{\text{Volume of sample}}$$

Water absorption and solubility indices of the instant flour were determined according to Yousf et al. (2017) and Atukuri et al. (2019). One gram of flour sample and ten milliliters of distilled water were combined in a centrifuge tube. The mixture was allowed to sit at room temperature for 30 min, stirring gently every 5 min, and centrifuged for 15 min at 3000 rpm (revolutions per minute). The supernatant liquid should be poured carefully into a tared evaporating dish. WAI was computed as grams of gel obtained per gram of substance after the residual gel was weighed (Equation 2). On the other hand, WSI was calculated as grams of dissolved solid in supernatant per gram of dry solid multiplied by 100 (Equation 3).

(2) $$\mathrm{WAI}\;\left(g/g\right)=\frac{\text{weight of sediment}\;}{\text{Weight of}\;\mathrm{dry}\;\text{solid}\;}$$

(3) $$\mathrm{WSI}\;\left(\%\right)=\frac{\text{Weight of dissolved solid in supernatant}*100}{\text{Weight of}\;\mathrm{dry}\;\text{solids}\;}$$

### 2.6 Determination of proximate composition

The AOAC (2005) methods were used to determine all proximate compositions. The moisture content was determined by drying the material at 105°C in an oven until a consistent weight was achieved (Method 925.09). Crude protein was extracted using the micro Kjeldahl method, which involved first digesting the sample with sulfuric acid (H_2_SO_4_) and then distilling it with sodium hydroxide (NaOH), an alkaline substance (Method 979.09). The amount of protein was then measured using a 6.25 nitrogen‐to‐protein conversion factor. Crude fat was determined using hexane extraction in a Soxhlet extraction system (Method 920.39). The crude fiber was determined as the combustible and insoluble organic residue left over following alkaline (NaOH) distillation and acid (H_2_SO_4_) digestion of the sample (Method 962.09). Ash content was measured as the amount of inorganic residue that remained after the sample was burned at 550°C, until the organic matter was no longer present (Method 923.03). Carbohydrate content was estimated by difference (Ojokoh et al., 2020).

### 2.7 Minerals (Ca, Fe, and Zn) analysis

The calcium, iron, and zinc in the samples were determined by the method of the Association of Official Organic Chemists (AOAC) (2005) by using atomic absorption spectrophotometry (AAS). About 2.5 grams of the sample was weighed and ash was obtained at 550°C for 5 h. It was treated with 7 mL of 6 N hydrochloric acid (HCl) to wet it completely and carefully dried on a hot plate. Fifteen milliliters of 3 N HCl was added and the dish was heated on the hot plate until the solution just boiled. Then it was allowed to cool and it was filtered through a filter paper into a 50 mL volumetric flask. Again 10 mL of 3 N HCl was added to the dish and heated until the solution boiled. Finally, it was cooled and filtered into the volumetric flask. For the determination of calcium, 2.5 mL of lanthanum chloride (LaCl_3_) (10% w/v) was added to both standard and samples. Using an atomic absorption spectrophotometer, a calibration curve was prepared by plotting the absorption or emission values against the concentration by using a series of standard solutions. Reading was taken from the graph, which depicted the concentration that corresponds to the absorption or emission value of the sample and the blank.

### 2.8 Determination of Beta‐carotene

The beta‐carotene content of the sample was measured according to Muchoki et al. (2005). In a 50 mL extraction conical centrifuge tube, 1 g of sample was weighed in duplicate and combined with 40 mL of acetone (high‐performance liquid chromatography grade). The samples were filtered using a Buchner funnel and suction after being centrifuged for 60 s. About 40 mL of petroleum ether was added to the acetone extract in a separating funnel. Distilled water was added gradually and without shaking along the neck wall to stop the creation of an emulsion. Subsequently, the two stages were divided and the lower watery layer was disposed of. The samples were rinsed three to four times with distilled water (about 200 mL) to extract any remaining acetone. The upper layer was then collected into a 50 mL volumetric flask and residual water was removed using an anhydrous sodium sulfate (Na_2_SO_4_) filter arrangement. Using an ultraviolet (UV)–visible spectrophotometer, the absorbance of the ethereal extract was measured at 450 nm.

### 2.9 Consumer acceptability test

Sensory analysis of porridge was done at Hawassa University College of Agriculture, School of Nutrition, Food Science and Technology central skills laboratory. The porridge samples were placed in identical containers and coded with three‐digit random numbers. The sensory acceptability in terms of color, aroma, mouthfeel, taste, and the overall acceptability was then evaluated by a panel (*n* = 50) using a 5‐point hedonic scale (where 1‐ dislike very much, 2‐ dislike, 3‐ neither like nor dislike, 4‐ like, and 5‐ like very much).

### 2.10 Ethical approval

Ethical approval was acquired from Hawassa University, School of Nutrition, Food Science and Technology Research Approval Committee. Furthermore, a consent form was prepared. Panelists were asked to complete the consent form before participating in the product testing.

### 2.11 Experimental design and data analysis

Completely Randomized Design (CRD) was used to determine the formulation effect on the functional properties, proximate composition, and mineral and beta‐carotene contents. The data collected for each parameter were analyzed using one‐way analysis of variance (ANOVA) procedure of JMP Pro Version 13 statistical software. The mean comparison was done using Tukey's (honestly significant difference (HSD)) test at *p* ≤ .05 level of significance.

## 3 RESULTS AND DISCUSSION

### 3.1 Functional properties

The functional properties, such as bulk density (BD), water absorption index (WAI), and water solubility index (WSI) of maize flour and composite instant flours, are presented in Table 2. The BD of this study's maize porridge flour and extruded instant flour ranged from 0.70 g/m to 0.74 g/mL. Compared to composite instant flours, maize flour (control) had significantly (*p* < .05) lower bulk density. Lupine addition might increase the quantity of fiber and protein in the mix (Adem et al., 2020). Also, the significant difference between the bulk density of maize flour and composite instant flour could be due to differences in starch content and particle size (Awuchi et al., 2019). The BD values of the product in this study were slightly higher than reported studies on the extrusion of different maize and legume‐based products (Adem et al., 2020). This higher BD may be attributed to the difference in blending ratios, crop types, and processing methods. The increment of bulk density from this research was in agreement with the findings by Adem et al. (2020) and Kindeya et al., 2021. The bulk density (BD) of a powder or flour depends on how closely individual particles are packed together and it plays an important role during mixing (Haile et al., 2016). It measures flour heaviness, indicates that the volume of the flour in a package will not excessively reduce during storage, and also determines the packaging requirements (Atukuri et al., 2019). The manufacture of confectioneries, such as cakes, sweet pastries, and cookies, benefits from an increase in flour bulk density (Kindeya et al., 2021). However, a decrease in bulk density causes a reduction in packaging and transportation costs (Atukuri et al., 2019). Particle size and flour density have an impact on bulk density, which is crucial in establishing the amount of packaging needed. The cost of packing decreases with decreasing bulk density (Akinjide Olubunmi et al., 2017; Otondi et al., 2020). The high bulk density of flour suggests that it is suitable for use in food preparations (Kindeya et al., 2021).

**TABLE 2:** Functional properties of ingredients and instant flours.

	BD (g/mL)	WAI (g/g)	WSI (%)
Flour types			
Maize	0.71 ± 0.01^b^	4.72 ± 0.07^a^	9.98 ± 0.15 ^c^
OFSP	1.04 ± 0.06^a^	4.09 ± 0.15^a^	23.58 ± 1.05^a^
Lupine	0.90 ± 0.00^a^	2.50 ± 0.28^b^	12.28 ± 0.22^c^
Moringa	0.95 ± 0.02^a^	2.28 ± 0.08^b^	20.91 ± 0.45^b^
Products			
P0	0.70 ± 0.02^b^	4.76 ± 0.01^a^	10.09 ± 0.08^d^
P1	0.72 ± 0.07^a^	4.16 ± 0.02^b^	11.63 ± 0.09^c^
P2	0.73 ± 0.08^a^	3.96 ± 0.04^c^	13.08 ± 0.29^b^
P3	0.74 ± 0.03^a^	3.84 ± 0.01^d^	14.16 ± 0.23^a^

*Note*: P0 = maize flour only, P1 = 50% maize, 35% orange‐fleshed sweet potato, 10% sweet lupine, and 5% moringa leaf powder, P2 = 45% maize, 30% orange‐fleshed sweet potato, 15% sweet lupine, and 10% moringa leaf powder, P3 = 40% maize, 25% orange‐fleshed sweet potato, 20% sweet lupine, and 15% moringa leaf powder. Mean values with the same column with different superscript letters are significantly different from each other (*p* < .05) and values are averages of duplicate readings (mean ± SD, *n* = 2).

Abbreviations: BD, bulk density; WAI, water absorption index; WSI, water solubility index.

The water absorption index (WAI) indicates the ability of flour to absorb water and depends on the availability of hydrophilic groups that bind water molecules and on the gel‐forming capacity of macromolecules (Mandge et al., 2014). Food materials' capacity to absorb water is occasionally linked to their starch level, which implies that mixes with higher water absorption could be helpful for bakery goods like bread, cakes, and cookies that need to be hydrated to enhance dough handling properties (Ndife et al., 2020). In this study, the WAI of the composite extruded instant flour including maize flour (control) ranged from 4.76% to 33.84%. Compared to composite instant flours, maize flour (control) had significantly higher (*p* < .05) WAI. Water absorption index decreased when the addition of orange‐fleshed sweet potato, sweet lupine flour, and moringa leaf powder increased. This might be due to the low concentration of available starch in lupine flour, which may influence the extent of starch gelatinization in the barrel and cause a reduced water absorption index in the product (Adem et al., 2020). On the other hand, the higher water absorption phenomenon of instant multigrain porridge could be associated with the denaturation of proteins at higher temperatures during processing (Mandge et al., 2014). However, similar to this study, Adem et al. (2020) also observed a decrease in WAI in the extruded product by the addition of lupine to maize‐based extruded snack food. Foods with a high water absorption index (WAI) absorb more water when cooking, resulting in bulky, low‐energy, and nutrient‐dense foods. On the other hand, low WAI values are preferred because they are suitable for producing food products with a high caloric density per unit volume (Atukuri et al., 2019).

The water solubility index determines the number of polysaccharides released from the granule upon the addition of excess water and it is used as a measure for starch degradation (Mandge et al., 2014; Yousf et al., 2017). The lower WSI suggests that alternative blends can result in less sticky extrudates, which could lead to goods with desirable physical characteristics (Otondi et al., 2020). WSI of the maize flour (control) and composite extruded instant flour ranged from 10.09% to 14.16% (Table 2). Compared to composite instant flours, maize flour (control) had significantly (*p* < .05) lower WSI. This might be due to the addition of orange‐fleshed sweet potato, sweet lupine, and moringa leaf powder. Lupine is relatively high in fiber, protein, and fat content (Kefale & Yetenayet, 2020). A high concentration of fiber molecules disrupts the continuous structure in extruded products and high fiber concentration also hinders elastic deformation during the extrusion process (Moraru & Kokini, 2003). WSI increased with a rise in the blending ratio. Similarly, Adem et al. (2020) also observed an increase in WSI in extruded products by the addition of lupine to maize‐based extruded snack food. The higher WSI indicates more degradation of starch (Mandge et al., 2014). The water solubility index increases during extrusion because of high‐molecular‐weight carbohydrates and proteins are hydrolyzed into simpler components and high WSI can be used to predict the ease of digestion (Atukuri et al., 2019).

### 3.2 Proximate composition

The proximate compositions, such as moisture, protein, fat, fiber, ash, carbohydrate, and energy contents of maize flour and composite instant flours, are presented in Table 3. The mean values of moisture content of the extruded flours were significantly (*p* < .05) different from that of the control sample. These results were slightly lower than the result reported by Bekele and Shiferaw (2020) who found the values to be in the range between 6.79% and 7.32%. This might be the result of the various processing techniques used to develop the instant flours, but Terefe et al. (2022) who found in the range between 5.45% and 9.45% results are comparable to those of this study. The decrease in moisture content could be due to the addition of moringa leaf powder (lower hygroscopic nature of moringa leaf powder) contributing to the decrease in moisture content. This finding agreed with the finding of Belay et al. (2022) who reported a decreasing moisture content as moringa powder level increased in the complementary food preparation from locally available grains. This shows that the formulated instant flour sample might have more extended shelf life than maize porridge flour sample. Low moisture contents of formulations are also convenient for the packaging and transportation of products (Gbenyi et al., 2016). The moisture content of the formulated instant flour in the present study is within the recommended limit for extruded flour to have a prolonged shelf life (Terefe et al., 2022). In dried food, a moisture content between 6% and 10% has been recommended to prolong the shelf life of foods (Asare et al., 2012).

**TABLE 3:** Proximate composition of maize, sweet lupine, OFSP, and moringa leaf powder and respective products.

	Moisture (%)	CP (%)	Ash (%)	CF (%)	CF (%)	CHO (%)	Energy (Kcal/100 g)
Flour types							
Maize	8.38 ± 0.42 ^a^	9.24 ± 0.05^c^	1.31 ± 0.41^c^	3.30 ± 0.14^b^	1.55 ± 0.07^c^	76.22 ± 1.10^b^	371.54 ± 2.91^b^
OFSP	4.12 ± 0.15^d^	5.20 ± 0.01^d^	2.07 ± 0.01^c^	1.23 ± 0.00^c^	3.92 ± 0.07^b^	83.44 ± 0.11^a^	365.71 ± 0.33^b^
Lupine	7.00 ± 0.01^b^	34.38 ± 0.69^a^	3.95 ± 0.07^b^	7.34 ± 0.16^a^	2.01 ± 0.07^c^	45.31 ± 1.01^d^	384.86 ± 0.15^a^
Moringa	5.98 ± 0.05^c^	19.83 ± 0.24^b^	8.07 ± 0.10^a^	3.24 ± 0.07^b^	10.90 ± 0.21^a^	51.97 ± 0.09^c^	316.38 ± 0.69^c^
Products							
P0	8.50 ± 0.08^a^	9.00 ± 0.00^d^	1.43 ± 0.07^c^	3.37 ± 0.02^d^	1.55 ± 0.05 ^d^	76.11 ± 0.22 ^a^	370.89 ± 0.70^b^
P1	6.14 ± 0.06^b^	15.51 ± 0.02^c^	2.65 ± 0.04^b^	4.01 ± 0.00^c^	2.56 ± 0.07^c^	75.25 ± 0.15^a^	399.19 ± 0.45^a^
P2	5.91 ± 0.10^bc^	18.21 ± 0.30^b^	2.81 ± 0.07^b^	4.38 ± 0.12^b^	2.79 ± 0.04^b^	71.79 ± 0.32^b^	399.52 ± 1.12^a^
P3	5.60 ± 0.04^c^	21.10 ± 0.02^a^	3.36 ± 0.08^a^	5.37 ± 0.07^a^	3.21 ± 0.01^a^	66.94 ± 0.02^c^	400.53 ± 0.65^a^

*Note*: P0 = Maize flour only, P1 = 50% maize, 35% orange‐fleshed sweet potato, 10% sweet lupine, and 5% moringa leaf powder, P2 = 45% maize, 30% orange‐fleshed sweet potato,15% sweet lupine, and 10% moringa leaf powder, P3 = 40% maize, 25% orange‐fleshed sweet potato, 20% sweet lupine, and 15% moringa leaf powder. Mean values within a column with different superscript letters are significantly different from each other at *p* < .05 and values are averages of duplicate readings (mean ± SD, *n* = 2).

Abbreviations: CF, crude fat; Cf, crude fiber; CP, crude protein.

The protein content of maize‐based instant porridge flour in the present study increased significantly (*p* < .05) from 9.00% to 21.10% when the proportion of sweet lupine, orange‐fleshed sweet potato, and moringa leaf powder in the composite increased. The lowest value (9.00%) was recorded for the control sample. The increase in protein content in the present study was much more dependent on the amount of sweet lupine flour and moringa leaf powder proportion in the composite instant flour. The highest crude protein content (21.10%) was obtained from a blend with 40% maize flour 25% OFSP, 20% sweet lupine flour, and 15% moringa leaf powder. This might be as a result of the blend (P3) having the upper bound of sweet lupine, which has the highest protein content. Similar to this study, high levels of sweet lupine flour supplementation in wheat flour were known to enhance the protein contents of composite flour of sweet lupine and wheat bread (Kefale & Yetenayet, 2020). Increased protein content in the instant porridge flour could also be attributed to moringa leaf powder addition, which consists of considerable amounts of proteins (Belay et al., 2022; Netshiheni et al., 2018). The result of this study agreed with Netshiheni et al. (2018) who reported a 10.0% to 21.2% crude protein increase in maize, moringa, and termite blend instant porridge. However, the result of the present study is higher than the reported value of 8.1% to 18.1% increase as a result of sweet lupine addition on extruded snack food from maize–lupine composite flours by Adem et al. (2020) and the reported value of 12.4% to 19.5% increase on porridge from OFSP, soybean, and moringa blends according to Gebretsadikan et al. (2015). The observed difference in the CP content of the product compared to those mentioned in other studies could be due to genetic factors, raw material used, and processing methods. The recommended protein requirement for complementary foods for adequacy in the products to combat protein malnutrition is 15%, suggesting the adequacy of protein in the products (Gbenyi et al., 2016).

Ash is a residue of organic matter remaining after heating to remove all water and organic matter and it is the measure of the total amount of minerals present in a food sample. The ash content of the developed products in the present study ranged between 1.43% and 3.36%. The formulations p1, p2, and p3 showed significantly (*p* < .05) higher ash content (2.65%, 2.81%, and 3.36%) than the control (1.43%). A significant (*p* < .05) increase in ash content was noted when OFSP, sweet lupine, and moringa leaf powder proportion increased. Particularly, moringa leaf powder and sweet lupines have contributed to higher ash contents in the blend because of their high mineral contents (Gebretsadikan et al., 2015). A similar result has been reported in sweet lupine fortified with wheat bread (Kefale & Yetenayet, 2020) and complementary food formulated from locally available grains and moringa leaf powder (Belay et al., 2022). The result of this study agrees with that of Belay et al. (2022) who reported (0.5%–3.8%) in grains and moringa blend. But, the result of the present study is higher than the reported value of (1.6%–2.2%) by Adem et al. (2020) for extruded snack food from maize–lupine composite flours and lower than the reported value (4.5%–4.9%) by Gebretsadikan et al. (2015) for a porridge from OFSP, soybean, and moringa blends. The observed difference in ash content of the product compared to those mentioned in other studies could be due to genetic factors, raw material used, and processing methods.

Dietary fats are important for maintaining good health, as they supply energy and carry fat‐soluble vitamins (A, D, E, and K) in the diet. They are also structural bodily components and are involved in vital physiological processes, including growth, development, and brain function (Gebrezgi, 2019). The fat content of the formulated instant flours in the present study is relatively higher than that of the maize flour (control). The fat content of the developed instant flour ranged between 3.37% and 5.37%, with the highest value observed in P3 (5.37%). There was a significant (*p* < .05) increase in the crude fat content of the formulated instant flour. Increase in fat content might be due to the possible increase in sweet lupine flour in formulated instant flour. A similar result has been reported in sweet lupine–wheat bread (Kefale & Yetenayet, 2020) and maize–soybean–moringa complementary food Gebrezgi (2019). The result of this study agrees with Adem et al. (2020) who reported 3.2%–5.7% fat content for maize and lupine blend extruded products. However, the results of this study are lower than Gebretsadikan et al. (2015) who reported 1.9%–8.2% for porridge from OFSP, soybean, and moringa. This might be due to the differences in crop types and the processing methods used during the development of the product. Low‐fat foods are good for shelf‐life stability because they are less prone to rancidity during storage (Kefale & Yetenayet, 2020; Terefe et al., 2022).

The crude fiber content of the extruded instant flour increased significantly (*p* < .05) from 2.56% to 3.21%. The lowest value (1.55%) was recorded for the control sample. The increase in fiber content could be due to the possible increase in moringa leaf powder and sweet lupine flour in formulated instant flour. The blend P3 had the highest crude fiber content (3.21%) and was found for the blend of 40% maize flour, 25% OFSP, 20% sweet lupine flour, and 15% moringa leaf powder. This result is in agreement with results of previous studies where high crude fiber content was associated with the addition of lupine and use of whole‐grain maize flour (Adem et al., 2020). Similarly, higher crude fiber contents were observed in moringa leaf powders than in OFSP (Gebretsadikan et al., 2015). The results of fiber in this study are higher than the results of Adem et al. (2020) who reported 1.6%–2.7% for extruded products from maize and lupine. However, the results of fiber in this study are lower than the results of Belay et al. (2022) who reported 2.6%–6.0% for a complementary food formulated from locally available grains and moringa. The observed difference in the fiber content of the product compared to other studies could be due to the differences in crop types and the processing methods used during the development of the product.

The carbohydrate (CHO) content of the formulated instant flour including the control ranged between 66.94% and 76.11%. There was a significant (*p* < .05) decrease in carbohydrate content in the formulation (P2 and P3) compared to control (P0). As the proportion of moringa leaf powder and sweet lupine flour increased, the carbohydrate content of the formulation decreased. The differences observed in CHO content in this study could be due to the low carbohydrate content of moringa leaf powder compared to cereals and legumes (Belay et al., 2022). A similar trend was observed when maize flour was supplemented with soybean and moringa leaves in the development of complementary food (Gebrezgi, 2019). It was also reported that increasing the level of OFSP in maize flour during flat‐bread preparation resulted in a decrease in the carbohydrate contents' final product (Tadesse et al., 2015; Terefe, 2015). The decrease in carbohydrate content of the instant porridge flour might be due to an increase in protein, fat, ash, and fiber contents, as the proportion of sweet lupine flours and moringa leaf powder in the formulation was increased because carbohydrate was measured by different methods. The current result agrees with the results of Kindeya et al., 2021, who found that biscuits made from wheat, orange‐fleshed sweet potato powder, and haricot bean composite flour had lower carbohydrate content. The result as regards CHO in this study also agrees with Adem et al. (2020) who reported 64.8%–81.4% for an extruded product from maize and lupine. However, the result of CHO in this study is higher than that by Belay et al. (2022) who reported 47.2%–56.2% for complementary food formulated from locally available grains and moringa. The observed difference in the CHO content of the product compared to other studies could be due to the differences in crop types and the processing methods used during development of the product.

The energy (calorific) values of the instant flours including control varied from 370.89 to 400.53 kcal/100 g. An increase in OFSP, sweet lupine flour, and moringa leaf powder proportion resulted in a significant (*p* < .05) increment in the energy value of the extruded product in the present study. This indicates that the higher sweet lupine and moringa leaf powder proportion in the composite can provide more energy value than maize flour porridge. This result is in agreement with those of previous studies where the increase in energy content was associated with the addition of moringa flour (Fausat Lola et al., 2017). Similarly, Belay et al. (2022) found that the formulation based on moringa had a much higher energy content than the raw and control formulations. Moreover, increasing the proportion of OFSP in the blends of maize, in the development of flatbread resulted in a decreased energy value of the final product (Terefe, 2015).

### 3.3 Minerals and beta‐carotene

The mineral composition and β‐carotene content of raw materials and composite instant flour samples are indicated in Table 4. The iron content of the extruded formulated instant flour increased significantly (*p* < .05) from 3.49 mg/100 g to 9.58 mg/100 g. The lowest value (3.49 mg/100 g) was recorded for the control sample. Increased iron content was obtained in the instant porridge flour when the proportion of moringa leaf and sweet lupine increased, which confirms previous findings that moringa contributes to iron content increment and can be used as a source of iron (Belay et al., 2022; Gebretsadikan et al., 2015). Iron is an important trace element for the normal functioning of the central nervous system and it also plays an important role in the formation of hemoglobin, a component of blood cells that transports oxygen in the bloodstream throughout the body (Gebretsadikan et al., 2015; Orisa & Udofia, 2019). The recommended daily allowance (RDA) value of iron for an adult is 5 mg/day (Ilelaboye, 2019). The iron content of the formulated instant porridge flour of this study is in agreement with the result by Belay et al. (2022) who reported content to be in the range of 7.2 mg/100 g to 18.6 mg/100 g. However, it was lower than the results by Gebretsadikan et al. (2015) who reported values to be between 16.9 mg/100 g and 21.6 mg/100 g. The observed difference in the iron content of the product compared to those mentioned in other studies could be due to genetic factors, different locations, and also processing methods (Al‐Zahrani, 2020; Jideani & Diedericks, 2014; Mikore & Mulugeta, 2017; Netshiheni et al., 2018).

**TABLE 4:** Mineral contents of ingredients and products.

	Fe (mg/100 g)	Ca (mg/100 g)	Zn (mg/100 g)	β‐Carotene (μg/g)
Flour types				
Maize	3.49 ± 0.28^c^	40.47 ± 0.29^c^	2.91 ± 0.01^b^	0.00 ± 0.00^c^
OFSP	0.73 ± 0.00^d^	39.33 ± 0.80^c^	0.39 ± 0.00^c^	122.74 ± 1.35^a^
Lupine	20.08 ± 0.58^a^	66.00 ± 1.41^b^	7.57 ± 0.45^a^	0.11 ± 0.00^b^
Moringa	8.01 ± 0.02^b^	440.25 ± 1.34^a^	2.73 ± 0.07^b^	1.59 ± 0.01^b^
Products				
P0	3.49 ± 0.02^d^	40.49 ± 0.42^d^	2.89 ± 0.01^c^	0.00 ± 0.00^c^
P1	7.66 ± 0.05^c^	106.96 ± 0.43^c^	3.29 ± 0.05^b^	45.35 ± 0.21^a^
P2	8.78 ± 0.05^b^	109.65 ± 0.84^b^	3.46 ± 0.00^ab^	43.05 ± 0.9^ab^
P3	9.58 ± 0.29^a^	113.34 ± 0.52^a^	3.62 ± 0.06^a^	41.00 ± 1.55^b^

*Note*: P0 = Maize flour only, P1 = 50% maize, 35% orange‐fleshed sweet potato,10% sweet lupine, and 5% moringa leaf powder, P2 = 45% maize, 30% orange‐fleshed sweet potato,15% sweet lupine, and 10% moringa leaf powder, P3 = 40% maize, 25% orange‐fleshed sweet potato, 20% sweet lupine, and 15% moringa leaf powder. Mean values within a column with different superscript letters are significantly different from each other at *p* < .05 and values are averages of duplicate readings (mean ± SD, *n* = 2).

Abbreviations: Ca, calcium; Fe, iron; Zn, zinc.

The calcium content of the extruded products in the present study was found to be between 40.49 mg/100 g and 113.34 mg/100 g. All blends of instant flour samples varied significantly (*p* < .05) in calcium content. An increase in calcium content was obtained in the instant porridge flour when the proportion of moringa leaf and sweet lupine increased and this agrees with previous findings that moringa is high in calcium (Netshiheni et al., 2018). Moreover, the calcium content of sweet lupine is high as reported by Kefale and Abrha (2018) and Uzoaga (2020)who reported values of 10.36 mg/100 g to 2.97 mg/100 g for an extruded product from blends of cassava, sweet potato, and plantain fortified with moringa. However, the result of calcium in this study was observed to be lower than the results reported by Kanthi et al. (2017) who reported 164.75 mg/100 g for the instant herbal porridge mix by incorporating moringa leaf. Nutritional variability in food can be affected by factors, such as variety, postharvest handling, agrogeological conditions, and processing (Netshiheni et al., 2018; Sengev et al., 2013; Uzoaga, 2020). Calcium is very important for immunity and bone health and it also aids blood clotting and muscle contraction (Kanthi et al., 2017; Katmawanti et al., 2021). Recommended daily allowance (RDA) of calcium for adults is (1100 mg/day) and for lactating mothers it is 1000 mg requirements (Tessera et al., 2015).

In the present study, the zinc content of maize porridge flour and formulated instant flour ranged between 2.89 mg/100 g and 3.62 mg/100 g. The zinc content varied significantly (*p* < .05) when the formulation proportion varied. The zinc content of the control sample was observed to be lower than those of the formulated instant porridge flour samples. An increase in zinc content was obtained in the instant porridge flour when the proportion of sweet lupine increased and this agrees with previous findings (Kefale & Abrha, 2018). The zinc content in this study was observed to be lower than the results reported by Netshiheni et al. (2018). Nutritional variability in food can be affected by factors, such as the application of different fertilizers, maturity stage, variety, postharvest handling, and processing (Netshiheni et al., 2018; Prasanthi et al., 2017). However, the zinc content in this study agrees with the results by Belay et al. (2022) who reported values in the range of 3.0 mg/100 g to 5.9 mg/100 g. Zinc is a mineral that is essential for many of the body's normal functions and systems, including the immune system, wound healing, and blood clotting.

Foods high in β‐carotene can increase Vitamin A (VA) intake and reduce the cause of Vitamin A deficiency (VAD). The beta‐carotene content of the extruded flour increased significantly (*p* < .05) from 0 μg/g to 45.35 μg/g. However, zero beta‐carotene content was observed only in the formulation containing 100% maize. The increase in β‐carotene content in the formulated instant porridge flour could be due to the OFSP flour and moringa leaf powder. This study's β‐carotene content is lower than the result found by Kindeya et al., 2021 who found the β‐carotene content to be in the range of 29.6 to 64.43 μg/g for biscuits made from wheat, haricot bean, and OFSP flour. The β‐carotene content in this study is slightly higher than the result found by Honi et al. (2018) who reported values to be in the range of 38.1 to 40.7 μg/g for extruded snacks made from orange‐fleshed sweet potato and Bambara groundnut flour. Even though β‐carotene content rises as OFSP content rises, the values vary from product to product. Since β‐carotene is susceptible to heat degradation, the explanation might be given due to varieties, growing conditions, stages of maturity, harvesting and postharvest handling, processing, and storage of OFSP (Kindeya et al., 2021). According to the World Health Organization (WHO), the daily recommended dietary allowance of VA for pregnant/lactating women and children (6–59 months) is 800 μg and 400 μg, respectively (Kindeya et al., 2021).

### 3.4 Consumer acceptability

**FIGURE 1:**
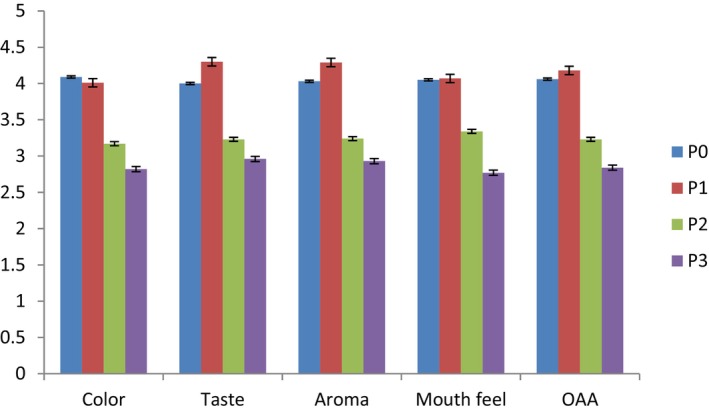
Sensory acceptability of formulated instant porridge.

Sensory evaluation results of instant flour porridge (P1) showed that the addition of OFSP flour, sweet lupine flour, and moringa leaf powder enhanced the taste and aroma of instant porridge (Figure 1). However, the addition of OFSP flour, sweet lupine flour, and moringa leave powder has significantly (*p* < .05) decreased the color, aroma, taste, mouth feel, and overall acceptability of instant porridge (P2 & P3). The degree of acceptability increased with an increase in the substitution level of OFSP flour in the formulation. This might be because the sweetness and the color of the sample's brightness increased with the increase in the proportion of OFSP flour, which could be a reason for its acceptability by panelists. The result of the current study is also in agreement with Tadesse et al. (2015) who reported that the acceptability of flat bread increased with the substitution of maize by orange‐fleshed sweet potato flour up to 35%. The degree of acceptability decreased with an increase in substitution level of sweet lupine flour and moringa leaf powder. This might be because of the leafy, herbal flavor, and dark green color of the moringa leaf powder caused by chlorophyll, which is also responsible for masking the color of foods when the inclusion is in large amount (Karim et al., 2013), which is different from the normal white color of maize‐based porridge. A similar report was also made by Netshiheni et al. (2018) where the addition of moringa leaf and termite powders increased the ratings for the color, taste, and aroma of the formulated maize‐based porridge that had decreased. In general, the results of the sensory evaluation showed that, instant porridge prepared from blends of 50% maize flour, 35% OFSP flour, 10% sweet lupine, and 5% moringa leaf powder had significantly higher acceptability than other blends in all attributes. Similar results were reported by Tadesse et al. (2015), Kefale and Yetenayet (2020), Netshiheni et al. (2018), Terefe (2015), and Gebretsadikan et al. (2015).

## 4 CONCLUSION

The results of this study showed that the addition of orange‐fleshed sweet potato, sweet lupine, and moringa leaf powder to maize flour for the development of instant porridge flour had led to a substantial increase in the nutritional quality of the final product. Protein, ash, fiber, beta‐carotene, iron, calcium, and zinc content of maize‐based instant porridge flour was improved. Therefore, it could be used as one of the sustainable strategies to alleviate malnutrition. It also showed that it is possible to obtain nutritionally enhanced instant porridge flour from maize–orange‐fleshed sweet potato–sweet lupine–moringa leaf composites using extrusion cooking. Instant porridge flour prepared from blends of 50% maize flour, 35% OFSP flour, 10% sweet lupine flour, and 5% moringa leaf powder was accepted more than the other blends in all attributes.

## AUTHOR CONTRIBUTIONS

**Tsiyonemariam Nega Dessta:** Conceptualization (equal); data curation (equal); formal analysis (equal); funding acquisition (equal); investigation (equal); methodology (equal); resources (equal); writing – original draft (equal). **Zemenu Kerie Terefe:** Conceptualization (equal); data curation (equal); formal analysis (equal); investigation (equal); methodology (equal); supervision (equal); validation (equal); visualization (equal); writing – original draft (equal); writing – review and editing (equal).

## CONFLICT OF INTEREST STATEMENT

The authors declare that they have no conflict of interest.

## Data Availability

All the supporting data are available and can be provided upon reasonable request.
